# Gut microbiota in children with type 1 diabetes differs from that in healthy children: a case-control study

**DOI:** 10.1186/1741-7015-11-46

**Published:** 2013-02-21

**Authors:** Mora Murri, Isabel Leiva, Juan Miguel Gomez-Zumaquero, Francisco J Tinahones, Fernando Cardona, Federico Soriguer, María Isabel Queipo-Ortuño

**Affiliations:** 1 Biomedical Research Laboratory, Virgen de la Victoria Hospital (FIMABIS), Campus de Teatinos s/n, Málaga, 29010, Spain; 2 Pediatric Endocrinology Service, Carlos Haya Materno Infantil Hospital, Avenida Arroyo de los Angeles, Málaga, 29011, Spain; 3 Molecular Biology Laboratory, Civil Hospital (IMABIS foundation), Plaza Hospital Civil s/n, Málaga, 29009, Spain; 4 CIBER of Physiopathology of Obesity and Nutrition (CIBEROBN), Instituto de Salud Carlos III, C/ Sinesio Delgado nº 4, Madrid, 28029, Spain; 5 Endocrinology and Nutrition Service, Carlos Haya Hospital, Plaza Hospital Civil s/n, Málaga, 29009, Spain; 6 CIBER of Diabetes and Metabolic Diseases (CIBERDEM), Instituto de Salud Carlos III, C/ Sinesio Delgado nº 4, Madrid, 28029, Spain; 7 Endocrinology and Nutrition Service, Virgen de la Victoria Hospital, Campus de Teatinos s/n, Málaga, 29010, Spain

**Keywords:** butyrate-producing bacteria, glycemic level, gut integrity, gut microbiota, gut permeability, HbA1c level, lactic acid-producing bacteria, mode of delivery, mucin, PCR-DGGE, type 1 diabetes

## Abstract

**Background:**

A recent study using a rat model found significant differences at the time of diabetes onset in the bacterial communities responsible for type 1 diabetes modulation. We hypothesized that type 1 diabetes in humans could also be linked to a specific gut microbiota. Our aim was to quantify and evaluate the difference in the composition of gut microbiota between children with type 1 diabetes and healthy children and to determine the possible relationship of the gut microbiota of children with type 1 diabetes with the glycemic level.

**Methods:**

A case-control study was carried out with 16 children with type 1 diabetes and 16 healthy children. The fecal bacteria composition was investigated by polymerase chain reaction-denaturing gradient gel electrophoresis and real-time quantitative polymerase chain reaction.

**Results:**

The mean similarity index was 47.39% for the healthy children and 37.56% for the children with diabetes, whereas the intergroup similarity index was 26.69%. In the children with diabetes, the bacterial number of Actinobacteria and Firmicutes, and the Firmicutes to Bacteroidetes ratio were all significantly decreased, with the quantity of Bacteroidetes significantly increased with respect to healthy children. At the genus level, we found a significant increase in the number of *Clostridium, Bacteroides *and *Veillonella *and a significant decrease in the number of *Lactobacillus, Bifidobacterium, Blautia coccoides/Eubacterium rectale *group and *Prevotella *in the children with diabetes. We also found that the number of *Bifidobacterium *and *Lactobacillus*, and the Firmicutes to Bacteroidetes ratio correlated negatively and significantly with the plasma glucose level while the quantity of *Clostridium *correlated positively and significantly with the plasma glucose level in the diabetes group.

**Conclusions:**

This is the first study showing that type 1 diabetes is associated with compositional changes in gut microbiota. The significant differences in the number of *Bifidobacterium, Lactobacillus *and *Clostridium *and in the Firmicutes to Bacteroidetes ratio observed between the two groups could be related to the glycemic level in the group with diabetes. Moreover, the quantity of bacteria essential to maintain gut integrity was significantly lower in the children with diabetes than the healthy children. These findings could be useful for developing strategies to control the development of type 1 diabetes by modifying the gut microbiota.

## Background

Type 1 diabetes is a worldwide problem, mainly in children, and it is associated with a significant burden, mostly related to the development of vascular complications [1]. Type 1 diabetes is the result of a complex interaction between different degrees of genetic susceptibility and environmental factors [2-4]. The intestinal microbiota is one of these environmental factors currently under study, partly as a result of observations in both non-obese diabetic (NOD) mice and BioBreeding diabetes-prone rats, where the use of antibiotics was shown to prevent the onset of diabetes [5,6]. Moreover, a recent study using NOD mice suggested that the development of type 1 diabetes can be prevented through modulation of the intestinal microbiota [7]. Newly, Vaarala *et al*. suggested that the interaction between the intestinal environment, the barrier function and the immune system are crucial in the onset of type 1 diabetes [4]. Using a rat model, Roesch *et al*. found significant differences at the time of diabetes onset in the bacterial communities responsible for type 1 diabetes modulation [8]. Moreover, other studies have shown that beneficial bacteria, such as probiotic bacteria, have a protective effect in rodent models by delaying or preventing the onset of type 1 diabetes [9,10]. With respect to mechanisms of action, Wen *et al*. found that the gut microbiome of NOD mice lacking an adaptor for multiple innate immune receptors responsible for recognizing microbial stimuli correlates with the disease onset, revealing a relationship between gut microbiota and the immune system [11]. Recent studies have demonstrated that commensal bacteria are crucial for maturation and function of the mucosal immune system. The balance between two major effector T cell populations in the intestine, IL-17+ T helper 17 cells and Foxp3+ regulatory T cells, requires signals from commensal bacteria and is dependent on the composition of the intestinal microbiota [12-14]. In addition, increased gut permeability has been observed in patients with type 1 diabetes as well as in NOD mouse and BioBreeding rat models [15-18]. It has been suggested that this increased gut permeability (commonly called leaky gut) may affect the absorption of antigens that can attack and damage pancreatic beta cells [19]. Because gut microbes can affect intestinal permeability, the gut ecology may play a role in the development of type 1 diabetes [20].

Only a few studies have evaluated the ecology of intestinal microbiota in autoimmune children who were not yet diabetic [21,22]. These studies used a very low number of participants (four patients and four controls) and neither of them have controlled for such an important factor as the mode of delivery (natural birth or Cesarean) or the type and time of infant feeding (formula-fed or breast-fed), both of which determine the gut microbial composition during infancy [23,24].

The aim of the present study, therefore, was to characterize the composition of fecal microbiota in children with type 1 diabetes as compared with children without diabetes (controlling for such factors as mode of delivery and breastfeeding time) using PCR-denaturing gradient gel electrophoresis (DGGE) and real-time quantitative PCR (qPCR) analysis. This was to determine whether there were significant differences in the gut microbiota composition between these groups and, if so, to quantify the differences and determine the possible relation of the gut microbiota of children with type 1 diabetes with their glycemic level.

## Methods

### Study participants and design

The case-control study included 16 Caucasian children with type 1 diabetes, aged 7.16 ±0.72 years, and 16 healthy Caucasian children, aged 7.48 ±0.87 years. Type 1 diabetes was diagnosed following the criteria of the American Diabetes Association [25,26] and the appearance of at least two persistent, confirmed anti-islet autoantibodies (insulin autoantibodies, glutamic acid decarboxylase autoantibodies or tyrosine phosphatase autoantibodies). The patients with diabetes were treated and monitored according to a standard medical protocol. Patients were excluded if they had any other acute or chronic inflammatory diseases or infectious diseases at study entry. The study participants received no antibiotic treatment, probiotics, prebiotics or any other medical treatment influencing intestinal microbiota during the 3 months before the start of the study. The selected healthy children were all type 1 diabetes autoantibody negative and they were matched to the children with diabetes for age, gender, race, mode of delivery and duration of breastfeeding. The parents of the patients and controls completed a structured interview to obtain the following data: health status, lifestyle aspects (such as living environment and physical activity) and dietary habit. The dietary intake patterns in patients and controls were determined from a food frequency questionnaire that allowed us to assess the consumption of groups of foods. The written guardian or parental consents of the children were obtained. The sampling and experimental processes were performed with the approval of the local Ethics Committee of Ciudad de Jaen hospital. Stool samples were collected by parents at home and delivered to the storage area for frozen storage at -80ºC within one hour [27].

### Anthropometric measurements

Body weight and height were measured according to standardized procedures [28].

### Laboratory measurements

Fasting venous blood samples were collected. The serum was separated in aliquots and immediately frozen at -80ºC. Serum biochemical parameters were measured in duplicate. Serum glucose, cholesterol and triglycerides were measured using a standard enzymatic method (Randox Laboratories Ltd., Antrim, UK). The quantitative detection of autoantibodies to islet cell antigens was done using the Elisa RSR GADAb Kit, Elisa RSR IA-2Ab Kit and RIA RSR IAA Kit (RSR Limited, Cardiff, UK).

### DNA extraction from fecal samples

Fecal samples were immediately kept after collection at -80°C and stored until analyzed. DNA extraction from 200 mg of stools was done using the QIAamp DNA Stool Mini Kit (Qiagen, Hilden, Germany) following the manufacturer's instructions. The DNA concentration was determined by absorbance at 260 nm (A260), and the purity was estimated by determining the A260 to A280 ratio with a Nanodrop spectrophotometer (Nanodrop Technologies, Wilmington, DE, USA).

### Analysis of fecal microbiota by PCR-DGGE

Fecal samples from each participant were examined by determining PCR-DGGE profiles as recently published by us [29]. The V2 to V3 regions of the 16S rRNA genes (positions 339 to 539 in the *Escherichia coli *gene) of bacteria in the fecal samples were amplified by primers HDA1-GC (5′-**CGC CCG CCG CGC GCG GCG GGC GGG GCG GGG GCA CGG GGG G**CC TAC GGG AGG CAG CAG T-3′; (the GC clamp is in boldface)) and HDA2 (5′-GTA TTA CCG CGG CTG CTG GCA C-3′) generating a 200 bp product. Aliquots (2 μL) of DNA were amplified by real-time PCR (20 μL final volume) in a 7500 Fast Real-Time PCR Systems instrument using Fast SYBR Green Master Mix and 200 nM of each of the universal primers HDA1-GC or HDA2 with the following amplification program: initial denaturation at 95°C for 20 s; amplification using 45 cycles including denaturation at 95°C for 3 s; annealing at 55°C for 30 s; and extension at 72°C for 1 min. Negative controls without a DNA template were included in each analysis.

After real-time PCR, 15 μL of products were mixed with 6 μL of loading dye before loading. Electrophoresis was performed with a DCode Universal Mutation Detection System instrument (Bio-Rad Laboratories, S.A, Madrid, Spain). Six percent polyacrylamide gels were prepared and electrophoresed with 1× TAE buffer prepared from 50× TAE buffer (2 M Tris base, 1 M glacial acetic acid, 50 mM ethylenediaminetetraacetic acid (EDTA)). The denaturing gradient was formed by using two 6% acrylamide (acrylamide to bisacrylamide ratio 37.5:1) stock solutions (Bio-Rad). The gels contained a 20% to 80% gradient of urea and formamide that increases in the direction of electrophoresis. Electrophoretic runs were in a TAE buffer (40 mmol/L Tris, 20 mmol/L acetic acid, and 1 mmol/L EDTA, pH 7.4) at 130 V and 60°C for 4.5 h. Electrophoresis was stopped when a xylene cyanol dye marker reached the bottom of a gel. Gels were stained with ethidium bromide (0.5 mg/L) for 5 min, rinsed with deionized water, viewed by UV transillumination and photographed with Gelcapture image acquisition software (DNR Bio-Imaging Systems Ltd, Mahale HaHamisha, Jerusalen, Israel). All the samples were analyzed on the same DGGE run to avoid the possible influence of variations in electrophoretic conditions between different runs. No band was observed in the negative controls. Similarities between banding patterns in the DGGE profile were calculated based on the presence and absence of bands and expressed as a similarity coefficient. Gels were analyzed using BioNumerics software (Applied Maths, Sint-Martens-Latem, Belgium). Normalized banding patterns were used for cluster analysis. The Dice similarity coefficient was used to calculate pairwise comparisons of the DGGE fingerprint profiles obtained. A similarity coefficient value of 100% indicates that DGGE profiles are identical while completely different profiles result in a similarity coefficient value of 0%. The unweighted pair group method with arithmetic mean algorithm was used for construction of dendrograms.

### Sequencing of selected bands from DGGE gels

Bands were excised from DGGE gels with a sterile razor, placed in 40 μL sterile water and incubated at 4°C for diffusion of DNA into the water. DNA were used in a second PCR with HDA1/2 primers without a GC-clamp (initial denaturation at 95°C for 20 s, followed by 45 cycles including denaturation at 95°C for 3 s, annealing at 55°C for 15 s and extension at 72°C for 10 s). Subsequently, the PCR products were directly cloned into pCR 4-TOPO (Invitrogen, Carlsbad, CA, USA) according to the manufacturer's instructions. Plasmid DNA was isolated from the cells using the Qiagen Mini Spin Prep Kit (Qiagen), and subjected to PCR (HDA1/2-GC) as earlier described. PCR products were diluted until 20 ng/μL, purified with ExoSAP-IT (USB Corporation, Cleveland, OH, USA) and sequenced in an ABI 3130 (Applied Biosystems, Inc., Foster City, CA, USA) using the BigDie-Kit-Standard. Nucleotide sequence data obtained were analyzed using MicroSeqID v2.1.1 software (Applied Biosystems).

### Microbial quantification by real-time qPCR

Specific primers targeting different bacterial genera were used to characterize the fecal microbiota by quantitative real-time qPCR (Table 1) [30-36]. Briefly, quantitative PCR experiments were performed with a LightCycler 2.0 PCR sequence detection system using the FastStart DNA Master SYBR Green kit (Roche Diagnostics, Indianapolis, IN, USA). All PCR tests were carried out in duplicate, with a final volume of 20 μL containing 1 μL of each fecal DNA preparation and 200 nM of each primer (Table 1). The thermal cycling conditions used were as follows: an initial DNA denaturation step at 95°C for 10 min; 45 cycles of denaturation at 95°C for 10 s; primer annealing at optimal temperature for 20 s; extension at 72°C for 15 s. Finally, melt curve analysis was performed by slowly cooling the PCRs from 95°C to 60°C (0.05°C per cycle) with simultaneous measurement of the SYBR Green I signal intensity. Melting-point-determination analysis allowed the confirmation of the specificity of the amplification products. Each participant's extracted DNA was subjected to a human β-globin PCR to ensure that amplifiable DNA was successfully extracted from the sample and to monitor for PCR inhibitors with the reaction conditions described previously [37]. The bacterial concentration from each sample was calculated by comparing the threshold cycle values obtained from the standard curves with the LightCycler 4.0 software. Standard curves were constructed for each experiment using serial 10-fold dilutions of bacterial genomic DNA (of known concentration) from pure cultures, corresponding to 10^1 ^to 10^10 ^copies per gram of feces.

**Table 1:** Primers used for real-time PCR

Target group	Oligonucleotide sequence (5′-3′)	Reference	Amplicon size (bp)
Bacteroidetes	CATGTGGTTTAATTCGATGAT	Guo *et al*. 2008 [30]	126
	AGCTGACGACAACCATGCAG		
*Bacteroides*	GAGAGGAAGGTCCCCCAC	Guo *et al*. 2008 [30]	106
	CGCTACTTGGCTGGTTCAG		
*Lactobacillus*	GAGGCAGCAGTAGGGAATCTTC	Delroisse *et al*. 2008 [31]	126
	GGCCAGTTACTACCTCTATCCTTCTTC		
*Fusobacterium*	CCCTTCAGTGCCGCAGT	Friswell *et al*. 2010 [32]	273
	GTCGCAGGATGTCAAGAC		
Firmicutes	ATGTGGTTTAATTCGAAGCA	Guo *et al*. 2008 [30]	126
	AGCTGACGACAACCATGCAC		
Actinobacteria	CGCGGCCTATCAGCTTGTTG	Stach *et al*. 2003 [33]	600
	CCGTACTCCCCAGGCGGGG		
*Bifidobacterium*	CTCCTGGAAACGGGTGG	Matsuki *et al*. 2004 [34]	550
	GGTGTTCTTCCCGATATCTACA		
*Prevotella*	GGTTCTGAGAGGAAGGTCCCC	Bekele *et al*. 2010 [35]	121
	TCCTGCACGCTACTTGGCTG		
*Enterococcus*	CCCTTATTGTTAGTTGCCATCATT	Rinttilä *et al*. 2004 [36]	144
	ACTCGTTCTTCCCATGT		
Proteobacteria	CATGACGTTACCCGCAGAAGAAG	Friswell *et al*. 2010 [32]	195
	CTCTACGAGACTCAAGCTTGC		
*Clostridium* Cluster IV	GCACAAGCAGTGGAGT	Matsuki *et al*. 2004 [34]	239
	CTTCCTCCGTTTTGTCAA		
*Blautia coccoides*-*Eubacterium rectale *group	CGGTACCTGACTAAGAAGC	Rinttilä *et al*. 2004 [36]	429
	AGTTTCATTCTTGCGAACG		
*Veillonella*	ACCAACCTGCCCTTCAGA	Rinttilä *et al*. 2004 [36]	343
	CGTCCCGATTAACAGAGCTT		
β-globin	GAAGAGCCAAGGACAGGTAC	Fredricks *et al*. 2007 [37]	270
	CAACTTCATCCACGTTCACC		

The different strains used were obtained from the Spanish Collection of Type Cultures (CECT) (*Bacteroides vulgatus *NCTC 11154, *Fusobacterium varium *NCTC 10560, *Enterococcus faecalis *CECT 184, *Enterobacter cloacae *CECT 194, *Clostridium perfringens *CECT 376) and the American Type Culture Collection (ATCC) (*Bifidobacterium bifidum *ATCC 15696, *Lactobacillus casei *ATCC 334D-5, *Prevotella intermedia *ATCC 25611D-5, *Ruminococcus productus *ATCC 27340D-5 and *Veillonella dispar *ATCC 17745). Standard curves were normalized to the copy number of the 16S rRNA gene for each species. For species for which the copy number of 16S rRNA operon was not published, the copy number was calculated by averaging the operon numbers of the closest bacterial taxa from the ribosomal RNA database rrnDB [38]. Negative controls containing all the elements of the reaction mixture except template DNA were performed in every analysis and no product was ever detected. The data presented are the mean values of duplicate real-time qPCR analyses. The amplification efficiency of the qPCR for all primer pairs was determined using the linear regression slope of a dilution series based on the following equation E = 10^(-1/slope)^. We found that for 13 primer pairs the efficiency ranged from 98% (E = 1.96) to 100% (E = 2) with slopes values in the range of -3.4 to -3.32.

### Statistical analysis

Results are expressed as mean values and standard deviations. The statistical analysis was performed with SPSS 15.0 software (SPSS Inc., Chicago, IL, USA). The sample size was calculated to obtain a difference in the mean bacterial number between the healthy children and those with type 1 diabetes of at least 2 × 10^5 ^copies per gram of feces. With a power of 80%, an alpha error of 0.05 and an estimated standard deviation between group of 1.13 × 10^5 ^copies per gram of feces (data obtained from Wu *et al*. [39]), six children were needed in each group. However, we increased the number of participants to 16 children and 16 controls. The bacterial copy number values were converted into logarithmic values before the statistical analysis. Given the low number of participants analyzed, the Mann-Whitney U test was used to check changes in bacterial number and biochemical variables between the two groups. The Spearman correlation coefficient was calculated to estimate the linear correlations between variables. A multivariate regression analysis was performed to identify individual bacteria as independent predictors for plasma glucose level. Statistical significance was set at a *P *value of <0.05. All data are presented in the text as the mean ± SD.

## Results

### Diet

The healthy children and those with diabetes all had similar physical activity and dietary habits. The analysis of the food frequency questionnaires showed no significant differences in the consumption patterns of rice, wheat, vegetables, fish or meat between the two study groups, although the children with diabetes had a fast carbohydrate restriction (foods made with white flour and refined sugar).

### Anthropometric and biochemical measurements

The anthropometric and biochemical variables of the healthy children and those with diabetes are shown in Table 2. Apart from the levels of glucose and HbA1c, which were significantly higher in the children with diabetes, no other significant differences were seen between the groups in the anthropometric and biochemical variables. In addition, because the healthy children and the children with diabetes were matched for breastfeeding time and mode of delivery, no significant differences were noted in these variables.

**Table 2:** Anthropometric and biochemical variables in healthy children and children with type 1 diabetes

	Healthy children	Children with type 1 diabetes	*P*
**N**	16	16	
**Male/female**	8/8	8/8	
**Vaginal delivery/Cesarean section**	12/4	12/4	
**Age of debut (months)**	N/A	24.62 ±1.52	
**Diabetes duration (years)**	N/A	4.84 ±1.79	
**Age (years)**	7.48 ±0.87	7.16 ±0.72	0.266
**Body mass index (kg/m^2^)**	16.35 ±0.82	16.57 ±0.95	0.489
**Weight (kg)**	25.15 ±2.12	24.88 ±1.98	0.712
**Height (cm)**	120.65 ±5.05	118.48 ±4.96	0.211
**Birth weight **	3.33 ±0.19	3.42 ±0.24	0.249
**Glucose (mg/dL)**	84.61 ±1.99	158.56 ±3.78	0.001
**HbA1c (%)**	4.47 ±0.21	7.63 ±0.43	0.001
**Triglycerides (mg/dL)**	56.54 ±6.43	56.93 ±4.92	0.849
**Cholesterol (mg/dL)**	162.84 ±11.46	164.89 ±9.76	0.590
**Breast feeding time (months)**	3.98 ±1.32	4.00 ±1.12	0.963

Values are presented as means ± SD. Relationships between both groups were analyzed using the Mann-Whitney U test. Values are significantly different for *P *<0.05.

### PCR-DGGE and bacterial band identification

Variations were found in the presence or absence (qualitative) and intensity (quantitative) of the bands between the healthy children and the children with diabetes in the host-specific fingerprints generated. DGGE band profiles showed differences in band richness between the two groups. Analysis of the diversity of the microbiota showed that the mean of the DGGE bands was 13.85 ±3.87 for the healthy children and 11.63 ±3.64 for the children with diabetes, though the difference was not significant. Some bands were seen in fingerprints from all the children (in different lanes but at the same position), indicating that specific species of the predominant microbiota were common to all the children.

**Figure 1:**
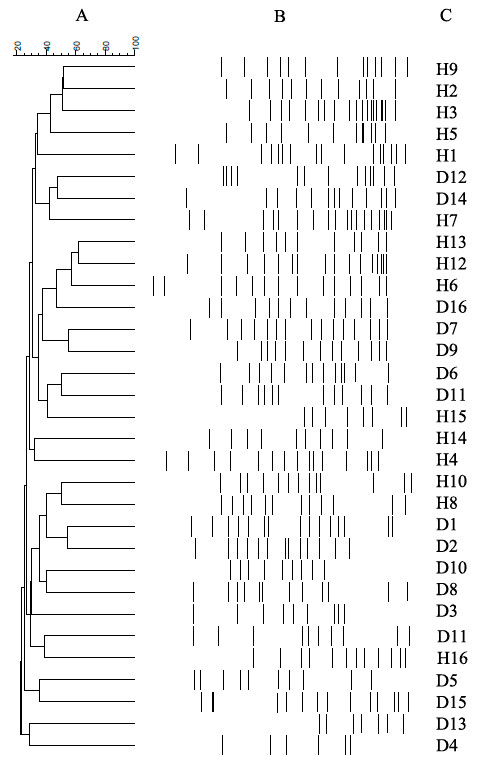
**Cluster analysis**. Dendrograms of electrophoretic band patterns obtained in the denaturing gel gradient electrophoresis experiment with universal primers in the fecal samples collected from healthy children (H) and those with type 1 diabetes (D). **(A) **Cluster analysis; **(B) **DGGE profiles related to fecal samples; **(C) **line graph.

The Dice similarity coefficient was used to calculate the similarity index between DGGE band profiles related to sampling of healthy children and those with diabetes. The mean similarity index was 47.39% for the healthy children and 37.56% for the children with diabetes. The mean similarity index between the groups was 26.69%, lower than the intra-group similarity (Table 3). The DGGE gel and the results of the cluster analysis are shown in Figure 1. The cluster analysis showed that the intra-group similarity for the diabetic and the healthy groups was significantly higher than the inter-group similarity. These results demonstrate that the dominant microbiota in the healthy group was different from that of the diabetic group.

**Table 3:** Microbiota diversity and similarity of healthy and type 1 diabetes groups of children

	Microbiota diversity	Microbiota similarity	
		
	DGGE bands^a^ (means ± SD)	Intra-group^b^	Inter-group^c^
**Healthy**	13.85 ±3.87	47.39 ±4.35	26.69 ±6.78
**Diabetic**	11.63 ±3.64	37.56 ±5.67	
***P***	0.105	0.001	0.000

Values are presented as means ± SD. Data were analyzed using the Mann-Whitney U test. Values are significantly different for *P *<0.05. ^a^Number of DGGE bands produced by each sample analyzed. ^b^Dice similarity coefficients comparing DGGE band profiles within individuals of the same group. ^c^Dice similarity coefficients comparing DGGE band profiles between members of the type 1 diabetes and the healthy groups.

All the bands from the profiles of all the healthy children and the children with diabetes were cloned and sequenced to identify the dominant microbiota. The sequence similarity matches for bands were analyzed by MicroSeqID v2.1.1 software. Bacterial identification showed that the majority of bacteria represented in the fingerprints obtained corresponded to five phyla (Table 4). Most of the sequences belonged to Firmicutes and Bacteroidetes, with the rest distributed among Actinobacteria, Fusobacteria and Proteobacteria. Nevertheless, we observed important differences between the healthy children and the children with diabetes in the distribution ratio of the different genera within Bacteroidetes, Firmicutes and Actinobacteria phyla. In the children with diabetes, we found an increase in the *Clostridium, Bacteroides, Veillonella, Eggerthella *and *Bacillus *frequencies and a disappearance of *Prevotella *and *Bifidobacterium *as compared with the healthy children (Table 4).

**Table 4:** Bacterial identification after sequencing the bands cloned from the DGGE analysis of fecal **samples in **the healthy and diabetic groups

Identification	Healthy group^a^ (n = 52)	Diabetes group^a^ (n = 50)	Sequence similarity (%)
**Phylum Firmicutes**			

*Veillonella*	4 (7.7%)	9 (18%)	99.97
*Bacillus*	0	2 (4%))	99.82
*Clostridium*	6 (11.5%)	15 (30%)	99.95
*Gemella*	4 (10%)	2 (4%)	99.86

**Phylum Fusobacteria**			

*Leptotrichia *	4 (7.7%)	3 (6%)	99.94

**Phylum Actinobacteria**			

*Bifidobacterium*	6 (11.5%)	0	99.75
*Eggerthella *	0	4 (8%)	99.90

**Phylum Proteobacteria**			

*Desulfovibrio*	4 (7.7%)	3(6%)	99.75

**Phylum Bacteroidetes**			

*Prevotella*	16 (30.7%)	0	99.68
*Bacteroides*	5 (9.6%)	10 (20%)	99.89
*Pedobacter *	3 (5.8%)	2 (4%)	99.47

^a^Refers to the frequency (and percent) of each unique bacteria genus in the type 1 diabetic group and healthy group. n refers to the number of bands cloned, sequenced and identified in each study group. N = 16 participants per group.

### Comparative analysis of gut microbiota communities in healthy children and children with diabetes

Changes in the bacterial population abundance were assessed in the fecal samples of both groups. The results obtained in the real-time qPCR experiments with the different primers are shown in Tables 5 and 6. Relevant differences were found in the bacteria number of three phyla between the diabetic and the healthy children. The number of Actinobacteria, Firmicutes and Bacteroidetes were significantly different between groups whilst the quantity of Proteobacteria and Fusobacteria were similar between the groups. In the children with diabetes, the bacterial number of Actinobacteria and Firmicutes was significantly decreased while that of Bacteroidetes was significantly increased with respect to the healthy children. Moreover, the Firmicutes to Bacteroidetes ratio was significantly lower in the children with diabetes than the healthy children.

**Table 5:** Real-time PCR quantification of microbiota phyla in healthy children and children with type 1 diabetes

	Healthy children	Children with type 1 diabetes	*P*
**Proteobacteria**	7.74 ±0.64	7.97 ±1.12	0.481
**Actinobacteria**	6.32 ±0.45	5.47 ±0.93	0.003
**Fusobacteria**	6.61 ±1.19	6.99 ±1.28	0.391
**Firmicutes**	9.85 ±0.43	6.54 ±0.56	0.001
**Bacteroidetes**	9.98 ±0.74	10.92 ±0.83	0.002
**Firmicutes to Bacteroidetes ratio**	0.97 ±0.19	0.62 ±0.24	0.001

Values are presented as means ±SD and expressed as log_10 _copies per gram of feces. N = 16 participants per group. Relationships between both groups were analyzed using the Mann-Whitney U test. Values are significantly different for *P *<0.05.

**Table 6:** Genera and groups within the phyla Firmicutes, Bacteroidetes and Actinobacteria in healthy children and children with type 1 diabetes

	**Healthy children**	**Children with type 1 diabetes**	***P***
***Prevotella***	10.95 ±0.57	9.03 ±0.99	0.001
***Clostridium***	4.85 ±0.34	6.87 ±0.52	0.001
***B. Coccoides-E rectale***	8.64 ±0.72	6.99 ±0.47	0.001
***Enterococcus***	5.80 ±1.35	5.94 ±1.21	0.760
***Veillonella***	6.76 ±0.82	8.93 ±1.12	0.001
***Bifidobacterium***	5.65 ±1.14	3.12 ±0.97	0.001
***Lactobacillus***	4.23 ±0.36	3.47 ±0.43	0.001
***Bacteroides***	8.64 ±0.45	10.67 ±0.63	0.001

Values are presented as means ±SD and expressed as log_10 _copies per gram of feces. N = 16 participants per group. Relationships between both groups were analyzed using the Mann-Whitney U test. Values are significantly different for *P *<0.05.

Within Firmicutes, the quantity of *Veillonella *was significantly higher and the number of bacteria from the *Blautia coccoides*-*Eubacterium rectale *group was significantly lower in the children with diabetes compared with the healthy children. The *Lactobacillus *number was significantly lower and *Clostridium *levels significantly higher in the children with diabetes. However, no significant differences were found in *Enterococcus *levels between the two groups. Within Bacteroidetes, the quantity of *Bacteroides *was significantly higher whereas the number of *Prevotella *was significantly lower in the children with diabetes compared with the healthy children. Finally, within Actinobacteria, the number of *Bifidobacterium *was significantly lower in the children with diabetes.

### Relationship between **gut **microbiota composition in children with type 1 diabetes and glycemic level

In the children with diabetes, we found a significant univariate correlation between the amount of specific bacterial groups and the plasma glucose levels (*Bifidobacterium *r = -0.797, *P *= 0.008; *Clostridium *r = 0.676, *P *<0.05; *Lactobacillus *r = -0.698, *P *<0.05; and Firmicutes to Bacteroidetes ratio r = -0.473, *P *<0.05) and HbA1c levels (*Bifidobacterium *r = -0.573, P <0.05; *Clostridium *r = 0.452, *P *<0.05; Firmicutes r = -0.559, *P *<0.05; Firmicutes to Bacteroidetes ratio r = -0.765, *P *= 0.012). A multivariate regression analysis that included all the bacterial groups analyzed showed that only the reduction in the number of *Bifidobacterium *and *Lactobacillus *was associated with the plasma glucose level (*P *<0.05, β = -0.476, R^2 ^= 0.587; and *P *= 0.012, β = -0.687, R^2 ^= 0.539, respectively) whereas the higher HbA1c level was associated with the decrease in the Firmicutes to Bacteroidetes ratio (*P *<0.001, β = -1.047, R^2 ^= 0.781) and the increase in the number of *Clostridium *(*P *= 0.016, β = 0.867, R^2 ^= 0.499).

## Discussion

In the present study we found significant differences in the fecal microbial composition between healthy children and children with type 1 diabetes. We are unaware of any other similar studies in children with type 1 diabetes using simultaneously DGGE molecular profiling, unweighted pair group method with arithmetic mean algorithm dendrogram construction, sequencing and real-time qPCR analysis. To determine the characteristics of the gut microbiota based on the condition of just type 1 diabetes, we excluded the influence of physiological factors such as age, gender, dietary habits and race. In addition, we also controlled for the mode of delivery at birth and the duration of breastfeeding. This was because the first year of life has a crucial impact on gut microbiota composition and epidemiological studies in humans at genetic risk for type 1 diabetes have suggested that a short duration of breastfeeding and early feeding in infancy with complex dietary proteins such as cow's milk proteins can modulate the development of beta cell autoimmunity, clinical type 1 diabetes, or both [40-42]. No significant differences were found between the two groups of children (type 1 diabetes and controls).

The DGGE analysis of the fecal microbiota revealed a significantly lower intra-group similarity index in children with diabetes than in healthy children. In other words, the DGGE profiles in healthy children were more similar to each other, whereas in children with diabetes they were less similar. A similar result was found by Giongo *et al*. [21]. These data suggest that diabetic status may influence specific bacterial groups of the gut microbiota community.

Sequence analysis of the DGGE bands cloned enable the association of specific bacterial genotypes with health or diabetes situations. Consistent with previous human and animal studies [11,21,39,37,43], the gut microbiota of healthy children and children with diabetes was predominately composed of Firmicutes and Bacteroidetes and the main difference lies in the proportion of genus-division bacteria within this two phyla and the Actinobacteria phylum between both the group with diabetes and the healthy group. These results suggest that the dominant microbiota genera are different in children with type 1 diabetes compared with healthy children. Recently, three robust clusters, referred to as "enterotypes", which are not nation or continent specific have been identified. Assignment of an individual microbiome into a given enterotype is based upon the relative enrichment of that microbiome in one of three genera: *Bacteroides *(enterotype 1), *Prevotella *(enterotype 2) or *Ruminococcus *(enterotype 3) [44]. In this study, within Bacteroidetes, the *Bacteroides *genus was prevalent in the diabetic group, whereas the *Prevotella *genus was associated with the healthy group. Thus, the type 1 diabetic gut microbiomes could be classified into enterotype 1 and the healthy microbiomes could be classified into enterotype 2.

As DGGE is considered a semiquantitative tool for monitoring the dynamics of the predominant bacterial species of fecal microbiota, an additional analysis with real-time qPCR was performed to obtain a quantitative estimation of the changes found in the gut microbiota between children with diabetes and healthy children. We noted significant quantitative differences between the major microbial phyla present in the feces of healthy children and those with diabetes. In contrast to the situation in healthy children, we found a significant increase in the quantity of Bacteroidetes and a significant decrease in the number of Firmicutes and Actinobacteria in children with type 1 diabetes. Our data showed a significantly lower Firmicutes to Bacteroidetes ratio in children with type 1 diabetes compared with healthy children. Moreover, we saw a negative correlation between this ratio and both the glucose and the HbA1C levels in children with diabetes, which could help to explain the significantly higher glycemic level in this group. In agreement with this, Giongo *et al*. observed that the Firmicutes to Bacteroidetes ratio in study participants with type 1 diabetes was changing during the first 6 months after birth before the development of the autoimmune disease. These authors showed a successive decline in Firmicutes and an increase in Bacteroidetes number in the gut microbiome over time until the children became diabetic [21]. Moreover, this imbalance observed at the phylum level between Bacteroidetes and Firmicutes has been previously described in several human disorders. A decline in the Firmicutes to Bacteroidetes ratio compared with controls has been described in human type 2 diabetes [45], whereas in Crohn´s disease, both Bacteroidetes and Firmicutes seem to decline [46]. The opposite happens in obesity, where the imbalance is due to the increase in the Firmicutes to Bacteroidetes ratio [47], indicating that obesity and diabetes are associated with different groups of intestinal microbiota.

However, the major difference between the two groups was found in the number of bacteria at genus-division level. The most remarkable result was the significant increase in the number of *Clostridium, Bacteroides *and *Veillonella *in the children with diabetes, whereas the number of *Lactobacillus, Bifidobacterium*, the *Blautia coccoides/Eubacterium rectale *group and *Prevotella *genus were all significantly decreased in children with diabetes. Our findings concerning the microbiota of children with diabetes are in line with observations in other animal studies. Roesch *et al*. found higher levels of *Lactobacillus *and *Bifidobacterium *in BioBreeding diabetes-resistant rats whereas *Bacteroides *and *Clostridium *were more abundant in BioBreeding diabetes-prone rats [27]. In contrast with this, however, Brown *et al*. found that *Lactobacillus *and *Bifidobacterium *were more abundant in participants with type 1 diabetes than in healthy participants [22].

The significant decrease in the number of *Lactobacillus *and *Bifidobacterium *observed in children with type 1 diabetes in our study was associated with their higher levels of plasma glucose, as indicated by the negative correlation found. Also, the regression analysis showed that the decrease in the number of *Lactobacillus *and *Bifidobacterium *could be associated with the plasma glucose level in the children with diabetes. In previous studies, the levels of *Bifidobacterium *have also been related to improved glucose metabolism, insulin resistance and low-grade inflammation [48,49]. Moreover, Valladares *et al*. determined that the administration of *Lactobacillus johnsonii *isolated from BioBreeding diabetes-resistant rats delays or inhibits the onset of type 1 diabetes in BioBreeding diabetes-prone rats [10].

Both *Lactobacillus *and *Bifidobacterium *have members with probiotic characteristics and these have been associated with positive effects for the host in the large intestine [50]. In addition, both bacterial groups have the capacity to produce the beneficial organic acid lactate, which is converted into butyrate by butyrate-producing bacteria in the gut [22]. Barcenilla *et al*. [51] showed that most of the butyrate-producing isolates from human fecal samples are related to the *Blautia coccoides-Eubacterium rectale *group. Previous studies have shown that butyrate induces mucin synthesis (a glycoprotein produced by the host that could maintain the integrity of the gut epithelium) [52], decreases bacterial transport across the epithelium [53], and improves gut integrity by increasing tight junction assembly [54]. In addition, the genera *Prevotella *are responsible for the degradation of this mucin [55]; thus, the significant decline in the numbers of the *Blautia coccoides-Eubacterium rectale *group and *Prevotella *that we found in children with type 1 diabetes compared with healthy children could indicate a reduction in mucin synthesis by the host and a lack of this mucin on the epithelial layer of the gut, which would lead to a significant alteration in intestinal permeability. Other studies have described an association between type 1 diabetes and compromised barrier permeability in humans and both the NOD mouse and BioBreeding rat models [16-18,20].

The significant increase in the number of *Clostridium, Bacteroides *and *Veillonella *in the children with diabetes with respect to the healthy children was accompanied by a significant positive correlation between both the plasma levels of glucose and HbA1c and the quantity of *Clostridium*. These bacteria are able to ferment glucose and lactate to propionate, acetate and succinate. However, these short fatty acids do not induce mucin synthesis [52]. This situation would, though, reduce the tight junction assembly, generating an increase in the gut permeability in children with type 1 diabetes [22].

Finally, we propose a possible mechanism to explain the relationship we have found between the gut microbiota present in children with type 1 diabetes and the glycemic levels observed. The short-chain fatty acids (such as butyrate and propionate) formed by this gut microbiota have a role in the regulation of the levels of gut hormones such as glucose-dependent insulinotropic polypeptide, glucagon-like peptide 1 and ghrelin. These hormones have important effects on carbohydrate metabolism [56], thus allowing gut microbiota to affect glycemic levels. In addition, Huml *et al*. have previously demonstrated an altered secretion pattern of gut hormones in children with type 1 diabetes that may impact on the metabolic control of diabetes in these patients [57]. Further studies will be necessary to demonstrate this proposed mechanism.

A limitation of the 16S rRNA gene-based method is that the function of the identified bacteria is unknown. Future studies using a microbial metagenomic sequencing analysis will be carried out to obtain information about the functional diversity of the bacterial community analyzed here.

## Conclusions

This is the first study showing that type 1 diabetes is associated with compositional changes in gut microbiota. Our results show that gut microbiota found in children with type 1 diabetes differed significantly from that found in healthy children. The gut microbiota in the children with diabetes was less similar than the gut microbiota in the healthy children. The significant differences between the diabetic and the healthy children in the number of *Bifidobacterium, Lactobacillus *and *Clostridium *and the Firmicutes to Bacteroidetes ratio could be implicated in the glycemic level of the children with diabetes. In addition, the numbers of lactic acid-producing bacteria, butyrate-producing bacteria and mucin-degrading bacteria, essential to maintain gut integrity, were significantly lower in the children with diabetes than the healthy children. These bacterial differences could be responsible for the altered gut permeability previously described in patients with type 1 diabetes. These findings could be useful for developing strategies to control the development of type 1 diabetes by modifying the gut microbiota.

## Abbreviations

bp: base pairs; DGGE: denaturing gradient gel electrophoresis; EDTA: ethylenediaminetetraacetic acid; NOD: non-obese diabetic mice; PCR: polymerase chain reaction; qPCR: quantitative polymerase chain reaction; SD: standard deviation; TAE: buffer with Tris base, glacial acetic acid and ethylenediaminetetraacetic acid.

## Competing interests

The authors declare that they have no competing interests.

## Authors' contributions

IL, FC, FS, FJT and MIQO conceived the study and developed the experimental design. IL, FS, FJT and MIQO were responsible for acquisition and selection of all samples utilized in this study. MM, IL, FC, JMGZ and MIQO performed all laboratory assays. MM, IL, JMGZ, FC and MIQO compiled the database and performed statistical analysis and data interpretation. MM, IL, JMGZ, FC, FS, FJT and MIQO wrote the paper. FS, FJT and MIQO provided critical revision. All the authors have read and approved the final manuscript.

## Pre-publication history

The pre-publication history for this paper can be accessed here:

http://www.biomedcentral.com/1741-7015/11/46/prepub
